# Glycated Haemoglobin (HbA_1C_) in Cardiac Surgery: A Narrative Review

**DOI:** 10.3390/jcm14010023

**Published:** 2024-12-24

**Authors:** Suvitesh Luthra, Laura Viola, Manoraj Navaratnarajah, David Thirukumaran, Theodore Velissaris

**Affiliations:** 1 Wessex Cardiothoracic Centre, Division of Cardiac Surgery, University Hospital Southampton NHS Foundation Trust, Southampton SO16 6YD, UK; 2 Academic Unit of Human Development and Health, Faculty of Medicine, University of Southampton, Southampton SO17 1BJ, UK

**Keywords:** glycated haemoglobin, cardiac surgery, HbA_1C_, clinical outcomes, diabetes

## Abstract

**Background:** Perioperative dysglycaemia in cardiac surgery is associated with poor outcomes. Glycaemic variability rather than glucose levels is a predictor of the length of an ICU stay, a rise in creatinine and acute kidney injury after cardiac surgery. Glycated haemoglobin (HbA_1C_) values correspond closely to average blood glucose levels and cut-off values can be used to define a diabetic and pre-diabetic status. These have been correlated with perioperative events. **Methods:** In this narrative review, MEDLINE (via PubMed) and the Cochrane Library were used to search for the effects of different preoperative HbA_1C_ levels on the postoperative outcomes after cardiac surgery. HbA_1C_ values correspond closely with average blood glucose levels and cut-off values can be used to define a diabetic and pre-diabetic status; these have been correlated with perioperative events. This narrative review discusses the role of HbA_1C_ in cardiac surgery. **Discussion:** The scientific data show controversial results: some systematic reviews and randomised control trials demonstrated that a high level of HbA_1C_ seems to be an indicator for postoperative complications in cardiac surgery; other studies dissented and reported that mortality and morbidity cannot be directly attributed to HbA_1c_ levels. **Conclusions:** The scientific community seems to be in general agreement that high levels of HbA_1C_ are prognostic markers of adverse outcomes post cardiac surgery, but it has also been proved that there could be multiple underlying factors contributing to them.

## 1. Introduction

Glycated haemoglobin (HbA_1C_) was first isolated by Huisman and Meyering in 1958 [1]. Its use for measuring glycaemic variability in diabetic patients was first proposed by Koenig et al. in 1976 [2]. HbA_1C_ specifically refers to the measure of the beta-N-1-deoxy fructosyl component of haemoglobin, which forms after binding between glucose and the exposed N-terminal valine end of the β chain of haemoglobin. This irreversible, non-enzymatic condensation reaction results in the formation of a Schiff base which undergoes base-catalysed isomerization (the Amadori rearrangement of aldose to ketose) to form 1-deoxyfructose. The nomenclature is based on the fractional order of haemoglobin subtypes on cation exchange chromatography (the order of fractions being HbA0, HbA1a, HbA1b and HbA_1C_). The fraction of HbA_1C_ within the red blood cell is a measure of the average glycaemic exposure during the life time of the cell (an average of 110 days) and reflects the average glycaemic levels over a 3-month period.

## 2. Materials and Methods

**Figure 1:**
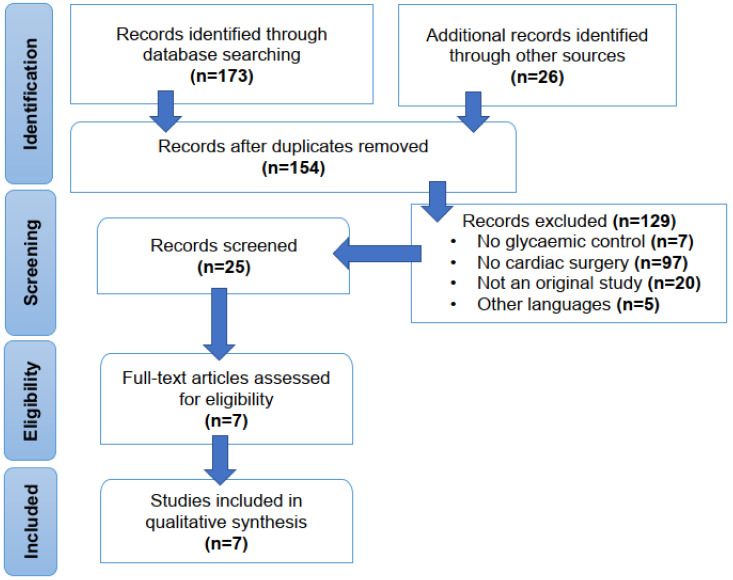
Preferred Reporting Items for Systematic Reviews and Meta-Analyses (PRISMA) chart of study selection.

A number of database resources, including MEDLINE (via PubMed), the Cochrane Library and Web of Science, were searched as per the Preferred Reporting Items for Systematic Reviews and Meta-Analyses (PRISMA) reporting guidelines for systematic reviews. The following keywords were used: HbA_1c_, glycated haemoglobin, cardiac surgery and diabetes. We also searched the references from original articles, narrative reviews, clinical guidelines and previous systematic reviews/meta-analyses to identify additional studies. We focused our search on the last five years, and we excluded studies with less than 1000 patients. The PRISMA flow chart for the search is shown in Figure 1.

## 3. Discussion

### 3.1. Representation and Values

HbA_1C_ can be expressed in older percentage units (DCCT, Diabetes Control and Complications Trial) or as mmol/mol SI units (IFCC, International Federation of Clinical Chemistry). Since 2007, the American Diabetes Association, European Association for the Study of Diabetes and the International Diabetes Federation have agreed to report values in IFCC units for the easier comparison of results and standards [3].

HbA_1C_ values correspond closely with average blood glucose levels, and cut-off values can be used to define a diabetic and pre-diabetic status (Table 1). An A_1C_-Derived Average Glucose (ADAG) study derived a mathematical relationship using a combination of continuous glucose monitoring and frequent finger stick capillary blood glucose testing in 507 subjects (including 268 subjects with type 1 diabetes, 159 subjects with type 2 diabetes and 80 subjects without diabetes) from 10 international centres. It established a validated relationship between HbA_1C_ and the average glucose across a range of diabetes types and patient populations [4,5,6,7].

### 3.2. Correlation with Blood Glucose Levels

The DCCT percentage (%) and eAG (estimated average glucose) measurement are given by the following equations [8,9]:

eAG (mg/dL) = 28.7 × A1C − 46.7

eAG (mmol/L) = 1.59 × A1C − 2.59, R^2^ = 0.84

### 3.3. Guidelines for Pre-Screening and Perioperative Management

The Joint British Diabetes Societies for Inpatient Care Group (JBDS-IP) was created in 2008 to suggest a set of diabetes inpatient guidelines and recommended standards of care, with the overall aim of improving inpatient care in secondary care organisations. It was created and supported by Diabetes UK, the Association of British Clinical Diabetologists (ABCD) and the Diabetes Inpatient Specialist Nurse (DISN) UK group, and works with NHS England, TREND-UK and with other professional organisations. The JBDS-IP has published a comprehensive set of guidelines for the referral, preoperative assessment, perioperative management and care after discharge of adults with diabetes undergoing surgery [10]. A summary of the JBDS-IP recommendations includes the following:

(a) Referrals for surgery must provide the current HbA_1C_, blood pressure and weight measurements with details of relevant complications and medications in the referral letter.
(b) Ensure that glycaemic control is optimised prior to surgery if safe to do so, aiming for an HbA_1C_ < 69 mmol/mol.
(c) Establish an individualised diabetes management plan, agreed with the patient, for the pre-admission and perioperative period.
(d) Referral to the diabetes specialist team according to local policy for all patients with hypoglycaemia unawareness and an HbA_1C_ > 69 mmol/mol (8.5%) where optimisation is safely achievable.
(e) The target blood glucose in the preoperative, anaesthetised or sedated patient should be 6–10 mmol/L (up to 12 mmol/L may be acceptable). The acceptable postoperative range in the awake patient is 6–12 mmol/L with a variable-rate intravenous insulin infusion and 4–12 mmol/L without the infusion.
(f) Safe discharge planning, patient education and communication with community teams to provide ongoing post-discharge support. HbA_1C_ levels to be checked every 3–6 months.

The JBDS-IP further sets institutional, National Patient Safety Agency (NPSA) and local audit standards to achieve these management goals.

The NICE (National Institute of Health and Clinical Excellence, UK) guidance for type 2 diabetes in adults (NG28, published December 2015, updated August 2019) focuses on patient education, dietary advice, managing the cardiovascular risk, managing blood glucose levels and identifying and managing long-term complications [11]. The recommendations include pre-screening for HbA_1C_ for all patients with a surgical referral. Known diabetes patients should have the HbA_1C_ test repeated if a test has not been performed in the last 3 months.

The Society of Thoracic Surgeons’ Practice Guideline Series for Blood Glucose Management During Adult Cardiac Surgery similarly recommends the routine use of HbA_1C_ tests for preoperative screening in cardiac surgery patients to guide perioperative glucose management (Class 1, LOE C) [12].

Adequate glycaemic control is associated with an HbA_1C_ < 7% (<5.3 mmol/mol and average blood glucose levels of <8.6 mmol/L).

### 3.4. Correlation with Outcomes in Cardiac Surgery

Diabetes is a significant condition modifier for coronary artery disease. It substantially affects the surgical management and outcomes after cardiac surgery [13,14]. A total of 80% of cardiac surgery patients have perioperative hyperglycaemia. Around 20% of these patients have ‘stress hyperglycemia’ and do not have impaired glucose tolerance or diabetes. HbA_1C_ differentiates these conditions as levels are normal in stress hyperglycaemia.

A high level of HbA_1C_ is believed to be an indicator for postoperative complications in cardiac surgery, despite the exact reasons for adverse outcomes with perioperative hyperglycaemia remaining unclear; putative mechanisms include free radical generation and injury from glycated haemoglobin and other proteins.

The NIHR-commissioned initial DARE (Database of Abstracts of Reviews of Effects) review from seven randomised control trials found that the incidence of early mortality following cardiac surgery was significantly reduced with tight glycaemic control (OR of 0.52, 95% CI of 0.30 to 0.91; three RCTs), and so was post-surgical atrial fibrillation (OR of 0.76, 95% CI of 0.58 to 0.99; five RCTs), the length of stay in the intensive care unit (MD of −0.57 days, 95% CI of −0.60 to −0.55; three RCTs), the duration of mechanical ventilation (MD of −3.69, 95% CI of −3.85 to −3.54; four RCTs) and the use of epicardial pacing (OR of 0.28, 95% CI of 0.15 to 0.54; three RCTs) [15]. There was significant statistical heterogeneity for early mortality (I^2^ = 71%), time spent in the intensive care unit (I^2^ = 99%) and the duration of mechanical ventilation (I^2^ = 94%). Perioperative dysglycaemia (both hyper- and hypoglycaemia) are deleterious. Glycaemic variability rather than glucose levels was a predictor of the length of the ICU stay, a rise in creatinine and acute kidney injury after cardiac surgery [16,17].

In a single-centre retrospective analysis of 3555 patients, an elevated HbA_1C_ level predicted in-hospital mortality after coronary artery bypass grafting (an odds ratio of 1.40 per unit increase, *p* = 0.019). An HbA_1C_ > 8.6% was associated with a 4-fold increase in mortality [18]. A significantly increased risk of myocardial infarctions and deep sternal wound infections was found for each unit increase. Complications such as renal failure (threshold of 6.7, odds ratio of 2.1), a cerebrovascular accident (threshold of 7.6, odds ratio of 2.24), and deep sternal wound infections (threshold of 7.8, odds ratio of 5.29) were more common in patients with elevated HbA_1C_.

Corazzari et al. [19], in a meta-analysis of thirty studies with 34,650 patients, found that lower levels of glycosylated haemoglobin reduced late mortality. Those with low preoperative glycosylated haemoglobin thresholds had the lowest risk of sternal wound infections (RR, 0.50; 95% CI, 0.32–0.80; *p* = 0.003 for HbA_1C_ < 7.5%). Those with an HbA_1C_ < 7% had a lower risk of stroke/transient ischaemic attack (risk ratio of 0.53; 95% confidence interval, 0.39–0.70; *p* < 0.0001), acute kidney injury (risk ratio, 0.65; 95% confidence interval, 0.54–0.79; *p* < 0.0001) and a reduced hospital stay [19].

A summary of the recent retrospective cohort studies on diabetes is provided in Table 2 [20,21,22,23,24,25,26].

Narayan et al., in their retrospective analysis of 4678 patients, found no difference in mortality rates between those with an HbA_1C_ < 6.5% (52.93%) and those with an HbA_1C_ > 6.5% (47.07%) (odds ratio, 1.36; 95% confidence interval [CI], 0.95 to 1.953; *p* = 0.08) [27]. Overall, an HbA_1C_ of 6.5% or higher was an independent risk factor for respiratory complications (odds ratio, 1.05; 95% CI, 1.008 to 4.631; *p* = 0.01) and sternal dehiscence (odds ratio, 2.161; 95% CI, 1.008 to 4.63; *p* = 0.04).

This association is also better established for high HbA_1C_ levels and late vascular and microvascular complications (including retinopathy) and late survival. The estimated glucose disposal rate (eGDR) is calculated from HbA_1C_ (24.31 − (12.2 × waist to hip ratio) − 3.29 × hypertension − 0.57 × HbA_1C_). The eGDR is a useful clinical marker for insulin resistance, which has been associated with an increased risk of coronary artery disease, microvascular complications and premature death [28]. In the SWEDEHEART registry, there was a significant association between eGDR and an increased risk of death (adjusted hazard ratio, 1.46 (95% CI, 1.12–1.90)) [29]. Similarly, Epstein et al. showed that patients with the lowest eGDR (most insulin-resistant), compared with the highest (least insulin-resistant), had a significantly greater risk of any diabetes complication (OR: 3.1; 95% CI: 1.2–8.1) [30]. Duncan et al. found that mortality and morbidity after cardiac surgery were significantly reduced when intraoperative hyper-insulinaemic normoglycaemia was achieved and concluded that providing exogenous glucose while targeting normoglycaemia may be preferable to simply normalising glucose concentrations [31].

Despite many studies suggesting that increased HbA_1C_ levels are indicators of adverse outcomes, some studies disagree and state that mortality and morbidity cannot be directly attributed to them. Although associations have been clear between high HbA_1C_ levels and wound infection rates, renal failure and perioperative ischaemic events, some studies have failed to establish a prolonged length of stay and increased perioperative mortality [31,32,33,34,35].

Van Den Boom et al. demonstrated in a large cohort study that there was no relationship between HbA_1C_ levels and mortality, reviewing the outcomes in 6393 patients undergoing cardiac surgery. The study found that HbA1c was not a significant predictor of postoperative mortality (*p* = 0.88) [36].

Further smaller studies similarly showed no significant difference in mortality following cardiac surgery according to any cut-off of HbA_1c_ levels considered [37].

There is conflicting clinical evidence of high HbA_1c_ levels as a prognostic marker of unfavourable outcomes after cardiac surgery., There is a consensus that there are multiple underlying contributing factors in addition to HbA_1C_ that have a significant role in unfavourable long term outcomes. These include hypertension, dyslipidaemia, hyper-homocysteinaemia and increased levels of C-reactive protein, oxidative stress and blood viscosity. Cardiac surgery, anaesthesia and the stress the patients are subjected to can exacerbate oxidative stress and increase blood viscosity. As a result, the effect of high HbA1c and the likelihood of developing a cardiovascular event are increased.

Therefore, future research is needed to further assess any possible clinical association.

### 3.5. Problems in Pre-Screening and Limitations in Cardiac Surgery

The utility of HbA_1C_ in cardiac surgery is limited to pre-screening and the monitoring of preoperative interventions only. It is not a reliable indicator of glycaemic therapy after a cardiopulmonary bypass as that is associated with Hageman factor activation, cell clumping and red cell destruction. Since the levels are based on the average life span of red blood cells, HbA_1C_ may not be a reliable indicator of glycaemic variability in anaemic patients, after blood loss, blood transfusions or valve-related haemolysis and in those with renal and liver disease.

Other limitations in testing the glycaemic variability with HbA_1C_ include patients with renal disease, who have higher HbA_1C_ levels as measured by ion exchange chromatography but normal levels with specific glycation tests; dietary modifications with multiple strains of probiotics that significantly reduce levels; and patients with sickle cell disease and haemoglobinopathies, who also have abnormal results.

Considering HbA_1C_ levels reflect the glycaemic exposure over a 3-month period and any significant change in them shows only after about 3 weeks from the start or intensification of glucose-lowering treatment, the utility is limited to only elective surgery patients on the waiting list, and there is a limited role of disease modification for risk amelioration in urgent and emergency cases. The levels have prognostic value and are predictive of postoperative outcomes. Referral patterns have changed, with greater emphasis on urgent rather than elective surgery in the last few years. The SCTS 2019-20 taskforce report showed a 50% decline in elective CABG referrals and a 33% increase in urgent CABG referrals over the last decade [12]. Further, most patients with chest pain, acute ischaemic events and shortness of breath cannot wait for months, even on elective cardiac waiting lists, for the optimisation of cardiac risk factors, including diabetes, based on the pre-screening results of HbA_1C_. Our own analysis of the pre-trial feasibility survey of the OCTOPUS trial for the preoperative optimisation of diabetes (based on HbA_1C_ pre-screening, a review of medications and advice on diet and exercise) among cardiac surgeons confirmed a lack of concern and engagement with diabetic teams despite the perceived and acknowledged risks of adverse outcomes in poorly controlled diabetic patients [38]. Despite guidelines and recommendations, unfortunately less than 50% of the units routinely pre-screened their patients for HbA_1C_ even among diabetics in the United Kingdom.

### 3.6. Point of Care Testing (POCT) and Assays

POCT is defined as rapid testing using portable instruments (typically with a small footprint) at the time of patient consultation. It provides immediate results for therapeutic decisions. Improved glycaemic control is achievable with fewer patient visits. POCT for HbA_1C_ has improved clinical outcomes in primary as well as secondary care settings, reduces visits, improves patient satisfaction and brings down costs in the management of diabetes [39,40,41,42]. It can also be used easily in remote and poorly accessible geographical areas. Trained personnel are not required for testing. POCT is less accurate than laboratory testing, and costs are higher. It is not recommended for the diagnosis of diabetes. The advantages and disadvantages are summarised in Table 3.

A number of kits are available for POCT which require 1–5 μL of freshly collected capillary blood from a finger prick or venous blood anticoagulated with heparin, ethylenediaminetetraacetic acid or fluoride oxalate [43]. The results are available within minutes after placing the blood on a test cartridge. Analysis is based on differences in either the structure or charge of glycated versus non-glycated haemoglobin. The imprecision for most devices is <3%.

Four types of assays are available:

(a) Cation exchange chromatography: Differences in the isoelectric point are used to separate haemoglobin species (HbA_1C_ and HbA_0_) by employing differences in ionic interactions between the cation exchange groups on the column resin surface and the haemoglobin in the blood sample.
(b) Immunoassay: The immunoassay method uses antibodies which bind to the N-terminal glycated tetra-peptide or hexa-peptide group of HbA_1C_, forming immune complexes which can be detected and measured using a turbidimeter or a nephelometer.
(c) Affinity chromatography: Affinity chromatography is a separation technique based on structural dissimilarities between glycated vs. non-glycated haemoglobin which utilises m-aminophenylboronic acid and its specific interactions with the glucose adduct of glycated haemoglobin.
(d) Enzymatic assay: The enzymatic quantification of HbA_1C_ is based on the cleavage of the beta chain of haemoglobin by specific proteases to liberate peptides, which then further react to produce a measurable signal.

POCT devices must meet criteria for quality and standardisation, which are set by national agencies and are targeted to the IFCC Reference Measurement Procedure (RMP). External quality assessment (EQA) data with a ‘real world’ perspective on method performance have been used, and more recently, sigma metrics have been applied alongside CLSI guidance [44].

POCT is invaluable in monitoring long-term glycaemic control, adjusting treatment and the quality of care and evaluating new therapies. It has also been used for screening and measuring the risk for diabetic microvascular complications.

### 3.7. Other Glycated Proteins and Assays

Other sugars in the blood like fructose and galactose bind to haemoglobin and other proteins in the blood in an irreversible non-enzymatic reaction, similarly to glucose. The avidity for haemoglobin and other blood proteins for fructose is much higher than for glucose. The assays are however more difficult for other protein–carbohydrate complexes. This assay is useful in conditions with an artificially lower HbA1c, such as anaemia, HIV and autologous blood donations.

Glucose can also bind non-enzymatically to albumin (glycated albumin), which has been used a glycaemic biomarker [45,46]. Because of a half-life of albumin of 10 days, glycated albumin reflects the glycaemic status and variability more rapidly than HbA_1C_ over a much shorter 2–3 weeks. It is not influenced by albumin levels as it is the ratio of the glycated to the non-glycated fraction. It is a more sensitive marker in those with a renal impairment and those on dialysis [47]. Unlike HbA_1C_, it is not affected by cardiopulmonary bypass, blood loss and transfusions in the perioperative period or renal impairment. Unlike HbA_1C_, glycated albumin does not show variations for different ethnicities and races. It has more potential than HbA_1C_ in elective and urgent cardiac surgery patients. Its use has, however, not been substantiated in cardiac surgery patients for preoperative optimisation and will need further trials [48,49]. Glycated albumin also has rapid POCT for pre-screening.

### 3.8. Other Glycaemic Biomarkers

Other potential glycaemic biomarkers include fructosamine and 1,5-anhydroglucitol (1,5-AG). 1,5-AG is a naturally occurring monosaccharide. Its levels decrease during hyperglycaemia > 180 mg/dL. Normal levels are reached approximately after 2 weeks in the absence of hyperglycaemia. It is therefore useful to identify glycaemic variability in patients with normal HbA1c and blood glucose. Its correlation with the average glucose levels and its prognostic significance are not as clear and POCT is not available.

## 4. Conclusions

HbA_1C_ remains an important pre-screening tool and reflects the immediate postoperative and long-term prognosis in cardiac surgery. Its utility may however be limited to elective cardiac surgery patients due to changing referral practices and caseload patterns in cardiac surgery units, with greater emphasis on urgent cases and expedited patient care pathways.

## Figures and Tables

**Table 1:** Correlation of HbA_1C_ with average blood glucose levels and diagnostic standards for diabetes in cardiac surgery.

HbA_1C_		Average Blood Glucose Levels		Diagnostic Standards for HbA_1C_ in Diabetes in Cardiac Surgery Patients
% Units (DCCT)	mmol/mol Units (IFFC)	mmol/L	mg/dL	
5	31	5.4 (4.2–6.7)	97 (76–120)	**NORMAL** <5.7% or 38.8 mmol/mol
6	42	7.0 (5.5–8.5)	126 (100–152)	
7	53	8.6 (6.8–10.3)	154 (123–185)	**PRE-DIABETES** 5.7–6.4% 38.8–46.4 mmol/mol
8	64	10.2 (8.1–12.1)	183 (147–217)	
9	75	11.8 (9.4–13.9)	212 (170–249)	
10	86	13.4 (10.7–15.7)	240 (193–282)	
11	97	14.9 (12.0–17.5)	269 (217–314)	**DIABETES** >6.5% or 47.5 moml/mol
12	108	16.5 (13.3–19.3)	298 (240–347)	
13	119	18.1 (15–21)	326 (260–380)	
14	130	19.7 (16–23)	355 (290–410)	
15	140	21.3 (17–25)	384 (310–440)	
16	151	22.9 (19–26)	413 (330–480)	
17	162	24.5 (20–28)	441 (460–510)	
18	173	26.1 (21–30)	470 (380–540)	
19	184	27.7 (23–32)	499 (410–570)	

HbA_1C_ levels expressed as percentage units (DCCT, Diabetes Control and Complications Trial) or as mmol/mol SI units (IFCC, International Federation of Clinical Chemistry).

**Table 2:** Table of studies.

	Study Details (Reference)	Study Design	*n*	Cohort	Main Findings	Conclusion
1.	Rodriquez-Quintero 2024 [20]	Retrospective cohort study	2424	Isolated coronary artery bypass surgery	Median age: 64 years (IQR of 57–71). Median bypass time: 95 min (IQR of 78–116). Median cross-clamp time: 78 min (IQR of 63–95). Incidence of severe AKI within 30 days: HbA_1C_ of <6.5% – 5.7%; HbA_1C_ of 6.5–8.5% – 6.7%; HbA_1C_ ≥ 8.5% – 9.1%. HbA_1C_ > 8.5% was independently associated with severe AKI (aOR of 1.59, 95% CI of 1.06–2.40). Severe AKI associated with increased 30d (aOR of 15.83, 95% CI of 7.94–31.56) and 90d mortality (aOR of 9.54, 95% CI of 5.46–16.67), prolonged length of stay (aOR of 3.46, 95% CI of 2.41–4.96), unplanned 30-day readmission (aOR of 2.64, 95% CI of 1.77–3.94) and increased all-cause mortality (aHR of 3.19, 95% CI of 2.43–4.17).	HbA_1C_ (≥8.5%) was independently associated with an increased 30-day risk of severe AKI, which is a consistent predictor of adverse outcomes after CABG. Delaying surgery to achieve optimal glycaemic control in an elective setting may be reasonable.
2.	Cooke 2023 [21]	Retrospective cohort study	2560	Coronary artery bypass surgery (on pump and off pump)	High HbA_1C_ for ONCABG: - New postoperative dialysis (*p* = 0.01); - Rates of readmission (*p* = 0.003); - Greater lengths of stay (*p* = 0.002). High HbA_1C_ for OPCABG: - Rates of operative mortality (*p* = 0.04); - Postoperative renal failure (*p* = 0.0001); - New postoperative dialysis (*p* = 0.0001); - Sternal wound infection (*p* = 0.01); - Greater lengths of stay (*p* = 0.03).	High levels of HbA_1C_ correlated with numerous adverse patient outcomes in both ONCABG and OPCABG. Differences were noted in which outcomes were most impacted between the two techniques. Preoperative diabetic medical optimisation is crucial to improve CABG outcomes in both on-pump and off-pump techniques.
3.	Deo 2021 [22]	Retrospective study (VA)	16,190	Isolated coronary artery bypass surgery	HbA_1C_ > 10% in youngest (mean age of 60.9 years) and had high rates of insulin dependence. Mortality increased with HbA1c > 8%, and especially with preoperative HbA1c levels > 9%. HbA1c of 8–10% and >10% was associated with increased MI (HR of 1.24 and HR of 1.39, respectively) and need for reintervention (HR of 1.20 and HR of 1.24, respectively).	A preoperative HbA_1C_ > 8% was associated with an increased risk of mortality and adverse cardiac events.
4.	Van den Eynde 2021 [23]	Retrospective cohort study	1774	Coronary artery bypass surgery (off pump)	Median follow-up—326 days. Sternal Wound Complications (SWCs): 10% (no diabetes) vs. 18% (diabetes) (*p* < 0.001). A higher HbA_1C_ was associated with the following: - Higher incidence of SWCs (OR of 1.24 per 1% increase, 95% CI: 1.04, 1.48; *p* = 0.016); - Higher grade of SWCs (OR of 1.25, 95% CI of 1.06, 1.48; *p* = 0.010). No association between glycaemia and incidence (*p* = 0.539) nor grade (*p* = 0.607) of SWCs. HbA_1C_ was associated with SWCs in diabetes patients younger than 70 years only (OR of 1.41, 95% CI of 1.17, 1.71; *p* < 0.001).	HbA_1C_ and glycaemia are associated with SWCs following OPCAB.
5.	Natarajan 2019 [24]	Prospective observational study	1080	All cardiac surgery with cardiopulmonary bypass	- 71.4% of patients with diabetes mellitus: HbA_1C_ > 7%. - In-hospital mortality of 6.3% (68/1080): 46 diabetic patients. - 70% of 46 diabetic patients: HbA_1C_ levels > 7%. Diabetic group with HbA_1C_ > 7%: - Longer ICU stay (4.19 ± 2.91 vs. 1.25 ± 3.37, *p* < 0.001); - Longer hospital stay (10.62 ± 3.74 vs. 9.71 ± 4.78); - Increased duration of mechanical ventilation (0.11 ± 0.31 vs. 1.15 ± 1.67, *p* < 0.001); - Mortality (OR: 1.057; 95% CI: 0.85–1.31; *p* = 0.62); - Renal failure (OR: 0.99; 95% CI: 0.99–1.00; *p* = 0.47).	Increased HbA_1C_ is associated with increased morbidity and mortality in patients undergoing cardiac surgery using cardiopulmonary bypass. Optimal preoperative HbA1c may improve the outcome following cardiac surgery.
6.	Khan 2019 [25]	Retrospective cohort study (Genesee County, MI)	1133	Isolated coronary artery bypass surgery	No difference in HbA_1C_ ≤ 7% and >7% in terms of the following: - Mortality rate (OR of 1.0, 95% CI of 0.4–2.3); - Composite of all infections (OR of 1.0, 95% CI of 0.7–1.6). Independently increased mortality with RF (OR of 5.9, 95% CI of 1.5–22.9), smoking (OR of 3.7, 95% CI of 1.3–11.2) or EF <35% (OR of 3.4, 95% CI of 1.4–8.3).	Although not different in controlled and uncontrolled diabetic groups, increased mortality associated with comorbidities like RF, smoking and congestive heart failure, which were highly prevalent.
7.	Robich 2019 [26]	Regional registry	6415	Isolated coronary artery bypass surgery (on pump)	Four HbA_1C_ groups: less than 5.7% (*n* = 1713), 5.7% to 6.4% (*n* = 2505), 6.5% to 8.0% (*n* = 1377) and more than 8% (*n* = 820). Higher HbA_1C_ values were associated with younger age, female sex, greater body mass index, more comorbid diseases, lower ejection fraction, more 3-vessel coronary disease, and recent myocardial infarction (*p* < 0.05 trend for all). HbA1c values were not associated with higher rates of in-hospital death or morbidity. Long-term survival was significantly worse as HbA1c increased. Risk of death increased by 13% for every unit increase in HbA1c (adjusted hazard ratio, 1.13; 95% confidence interval, 1.07 to 1.19; *p* < 0.001).	HbA1c is predictive of long-term survival, with higher levels associated with poorer prognosis.

**Table 3:** Point of care testing for HbA1c in diabetes—advantages and disadvantages.

Advantages	Disadvantages
Immediate results; Single visit at site of diabetes care; Easy with a finger stick sample; Trained phlebotomist/laboratory personnel not required; Useful for low-access populations with no laboratories; Almost as good as laboratory testing.	No evaluation of performance of testing; No standardisation; Less accurate than laboratory testing; Greater variability in test results; Cannot be used for diagnosis due to concerns about accuracy.
